# Low Perceived Social Support Is Associated with CD8+CD57+ Lymphocyte Expansion and Increased TNF-**α** Levels

**DOI:** 10.1155/2014/635784

**Published:** 2014-04-27

**Authors:** Alfredo Copertaro, Massimo Bracci, Nicola Manzella, Mariella Barbaresi, Benedetta Copertaro, Lory Santarelli

**Affiliations:** ^1^Healthcare Workers Service, ASUR, Area Vasta No. 2, 60025 Loreto, Italy; ^2^Occupational Medicine, Department of Clinical and Molecular Science, Polytechnic University of Marche, Torrette, 60020 Ancona, Italy

## Abstract

Social support has been supposed to have a positive impact on the function of the immune system. However, the relationship between perceived social support and immune function has not yet been fully investigated. In this cross-sectional study, we investigated the link between perceived social support and lymphocyte subpopulations and cytokines. 232 healthy subjects provided a blood sample and completed the Multidimensional Scale of Perceived Social Support (MSPSS) questionnaire. Lymphocyte immunophenotypes and cytokines were determined. Significantly increased CD8+CD57+ lymphocytes and TNF-**α** levels were found in group with low perceived social support. Multivariate linear regression corrected for possible confounders confirmed a significant role of perceived social support in predicting the number of CD8+CD57+ lymphocyte and TNF-**α** levels. This study supports the association between perceived social support and immune function. In particular, poor social support may be related to a state of chronic inflammation sustained by CD8+CD57+ lymphocyte expansion and increased TNF-**α** levels.

## 1. Introduction

Social support from various sources, such as family, friends, organizations, and coworkers, has been linked to many benefits for both physical and mental health [1–3]. Perceived social support refers to the insight that support would be available if needed. It is well documented that perceived social support has not only a direct effect on health but also an indirect one by buffering stress [4]. Epidemiological studies indicate that individuals with low levels of social support have higher mortality rates, especially as a result of cardiovascular disease [5–7]. Although more research is needed, there is also evidence linking support to lower cancer and infectious disease mortality [8–10].

Psychological stress and negative emotions that could be a consequence of low social support can also have an impact on the immune system [11–14].

Furthermore, alterations in immune activity may be related to increased morbidity and mortality in populations exposed to adverse psychosocial factors such as poor social networks, low socioeconomic status, and portrayed psychological distress [15–17].

Little is currently known about the direct effects of perceived social support on lymphocyte subpopulations and cytokines.

In this cross-sectional study, we investigated the link between perceived social support and immune parameters, including lymphocyte immunophenotypes and cytokines, in a group of healthy subjects.

## 2. Materials and Methods

### 2.1. Participants and Sampling

Participants in the study were nurses from the local National Health Service Hospital Unit, Ancona, Italy. The study was carried out in accordance with the Declaration of Helsinki's ethical standards. Being part of standard occupational health surveillance it needed no formal approval by the local ethics committee, which was nevertheless consulted and which granted an informal authorization. Inclusion criteria were age <60 years, no infectious disorders or chronic medical diseases, no history of major psychiatric disorders, and no current or recent treatment with drugs affecting the immune system (e.g., corticosteroids, cytostatics, immunosuppressors, and immunomodulators). 232 nurses, meeting the selection criteria, agreed to participate in the study and gave their written informed consent. They were asked to refrain from exercising, smoking, eating, drinking alcohol, or taking medications for at least 12 h before sampling.

A blood sample was collected between 08:30 and 09:30 am. Blood was analyzed for leukocyte count, immunophenotype, and cytokines. After blood collection, the nurses completed a self-administered standardized questionnaire on lifestyle and habits. Perceived social support was evaluated by the validated 12-item Multidimensional Scale of Perceived Social Support questionnaire (MSPSS). It is scored on a 7-point scale, where higher scores indicate greater perceived availability of support from family, friends, and other sources [18].

### 2.2. Laboratory Analysis

#### 2.2.1. Immunophenotype Analysis

Immunophenotype analysis was performed on fresh whole blood within 2 h of collection using a direct immunofluorescence cytofluorimetric assay. Flow cytometric acquisition was performed on FACScalibur using MultiSet software (Becton Dickinson, San Jose, USA). At least 10,000 events for each sample were acquired. The proportions of cells expressing CD19+ (B cells), CD3+CD4+ (T helper lymphocytes), CD8+ (cytotoxic/suppressor T lymphocytes), CD8+CD57+ (T lymphocytes with cytotoxic activity), and CD3−CD16+CD56+ (NK cells) were calculated. Absolute values were obtained based on lymphocyte counts provided by an automated Haematology Analyzer (Gen-S, Beckman-Coulter, Fullerton, CA, USA).

#### 2.2.2. Cytokine Analysis

Proinflammatory cytokines (IL-1*β*, IL-6, INF-*γ*, and TNF-*α*) were analyzed using a multiplex sandwich ELISA (SearchLight, Pierce Biotechnology, Rockford, IL, USA) according to the manufacturer's instructions. Each sample was tested in duplicate. Results are expressed as pg/mL.

### 2.3. Statistical Analysis

The values of continuous variables were expressed as median and 25th–75th percentile while ordinal variables were expressed as percentages. The participants' MSPSS score was classified into two categories using the median value as the cutoff. The Mann-Whitney* U* test for continuous variables or the Chi-square test for dichotomous or categorical variables was used to evaluate differences between low and high MSPSS score groups. In order to protect against Type I errors, differences in studied parameters were evaluated with the Bonferroni-corrected Mann-Whitney* U* test. Rho Spearman's correlation was applied to analyze the relationship between continuous parameters. Multivariate linear regression analysis was used to study the association between the MSPSS score and immune parameters, adjusting for sociodemographic characteristics. All the tests were two-tailed. A probability *P* < 0.05 was considered statistically significant. Data analysis was performed with SPSS 19.0 for Windows (SPSS Inc., Chicago, IL, USA).

## 3. Results

The sociodemographic characteristics of the 232 participants stratified into 2 groups by MSPSS score are reported in Table 1. The low MSPSS score group showed a significantly higher age (median 40.0 versus 37.0 years), job seniority (15.0 versus 14.0 years), and BMI (23.0 versus 22.0 Kg/m^2^) and a higher prevalence of alcohol drinkers (40.4% versus 25.0%) compared to the high MSPSS score group. The lymphocyte subpopulation and cytokine values are reported in Table 2; no pathological value was found among the participants. Significantly increased CD8+CD57+ lymphocytes (median 163 versus 110 cells/mm^3^) and TNF-*α* levels (median 22.6 versus 12.8 pg/mL) were found in the low MSPSS score group compared to the high MSPSS score group.

CD8+CD57+ lymphocytes were negatively correlated with the MPSS score (*P* < 0.05) and positively correlated with CD8+ lymphocytes, CD3−CD56+CD16+ lymphocytes, and TNF-*α* (*P* < 0.05) (Table 3). TNF-*α* was negatively correlated with the MSPSS score and positively correlated with CD8+CD57+ lymphocytes and all the studied cytokines.

Multivariate linear regression showed a significant role of MSPSS score in predicting the number of CD8+CD57+ lymphocytes (*β* = − 0.17, *P* < 0.05) and TNF-*α* (*β* = − 0.21, *P* < 0.05) (Table 4).

## 4. Discussion

Several studies have pointed out that a poor perceived social support may not only affect emotional well-being but also result in disease development [19, 20]. Chronic and/or traumatic stress owing to low perceived social support may lead to a downregulation of the immune function. On the other hand, a good perceived social support is associated with a reinforcement of immunity [21]. Recently, Carroll and colleagues found that low social support was related to shorter leukocyte telomere length in older people, in agreement with the hypothesis that the social environment may contribute to cellular aging [22]. A possible effect of social support-induced chronic stress on alterations in cellular aging may be explained by polyclonal expansions of T lymphocytes CD8+CD57+ representing a marker of immune senescence. CD8+CD57+ lymphocytes are activated cytotoxic T lymphocytes at a terminal stage of their differentiation with evidence of immunological senescence and limited proliferative capacity [23–25]. The CD57 antigen is normally expressed only by a minority of human CD8+ T lymphocytes, but its expression increases during chronic immune activation [25]. There is growing evidence that the CD8+CD57+ population plays a significant role in various diseases associated with chronic immune activation such as cancer, chronic intracellular infections, and physiological age-related changes in the immune system status [23]. Our results show that a low level of social support is related to an expansion of CD8+CD57+ lymphocytes.

An increase in TNF-*α* levels was found in the low MSPSS score group compared to the high MSPSS score group. TNF-*α* is a key cytokine, which plays a critical role in tumor immunity as well as in immunity against infection. It is produced mainly by activated macrophages, although it can be produced by many other cell types, such as lymphocytes, NK cells, and neurons [26]. The primary role of TNF-*α* is in the regulation of immune cells. The deregulation of TNF-*α* production has been implicated in infectious disease [27–29] and in a variety of human diseases including cancer [30–32], Alzheimer's disease [33], major depression [34], and inflammatory bowel disease [35]. TNF-*α* is deleterious to the host with regard to coping with autoimmune or chronic inflammatory diseases [36–38].

Although it has been reported that CD8+CD57+ lymphocytes may produce proinflammatory cytokines, such as TNF-*α* [39–42], our data indicate only a correlation between CD8+CD57+ lymphocytes and TNF-*α* levels without providing evidence of a direct relationship between these two immune parameters. Since CD8+CD57+ lymphocytes and/or TNF-*α* may sustain a state of chronic inflammation [23, 37], poor social support should be taken into account in patients with autoimmune or chronic inflammatory diseases.

## Figures and Tables

**Table 1:** Socio-demographic characteristics of the MSPSS groups according to low (<65.0) and high (≥65.0) MPSS score.

	Low MSPSS score	High MSPSS score
Age (years): median (25th–75th percentile)	**40.0 (33.0–44.0)**	37.0 (34.0–40.0)*
Gender (%): (male/female)	18.4/81.6	22.4/77.6
Job seniority (years): median (25th–75th percentile)	**15.0 (10.0–22.0)**	14.0 (8.0–17.8)*
BMI (Kg/m^2^): median (25th–75th percentile)	**23.0 (21.8–25.0)**	22.0 (21.0–24.0)*
Subjects taking physical exercise (%)	27.2	23.3
Shift-working nurses (%)	49.4	54.9
Alcohol drinkers (%)	**40.4**	25.0*
Smokers (%)	43.0	44.0
Marital status (%)		
Unmarried	21.1	31.0
Cohabiting	5.3	5.2
Married	70.2	60.3
Divorced	3.5	3.4
Subjects with offspring (%)	66.7	60.3

*P < 0.05 high versus low MSPSS score. Mann-Whitney analysis (continuous variables) or Chi-square test (dichotomous or categorical variables).

**Table 2:** Immune and cortisol values in the MSPSS groups according to low (<65.0) and high (≥65.0) MPSS score. Results are expressed as median (25th–75th percentile).

	Low MSPSS score	High MSPSS score
*Lymphocyte subpopulation *		
Total Lymphocytes (cells/mm^3^)	2164 (1778–2644)	2298 (1915–2661)
CD19+ lymphocytes (cells/mm^3^)	269 (185–358)	269 (193–351)
CD3+CD4+ lymphocytes (cells/mm^3^)	1077 (866–1273)	1159 (897–1425)
CD8+ lymphocytes (cells/mm^3^)	558 (443–795)	596 (490–686)
CD8+CD57+ lymphocytes (cells/mm^3^)	**163 (95**–**222)**	**110 (73**–173)*
CD3−CD56+CD16+ lymphocytes (cells/mm^3^)	215 (164–300)	201 (137–283)
*Cytokines*		
IL-1*β* (pg/mL)	0.6 (0.2–2)	0.7 (0.2–1.7)
IL-6 (pg/mL)	1.3 (0.7–4.3)	1.5 (0.9–4.2)
TNF-*α* (pg/mL)	**22.6 (7.5**–**46.1)**	**12.8 (6.0**–23.4)*
INF-*γ* (pg/mL)	2.6 (1.6–3.9)	2.7 (1.3–3.8)

*P < 0.05 high versus low MSPSS score (Bonferroni-corrected Mann-Whitney *U* test).

**Table 3:** Rho Spearman's correlation between CD8+CD57+ and TNF-*α* with MSPSS score and immune parameters.

	CD8+CD57+ Lymphocytes (rho)	TNF-*α* (rho)
MSPSS score	−0.229*	−0.269*
*Lymphocyte subpopulation *		
Total Lymphocytes	0.129	−0.016
CD19+ lymphocytes	−0.102	0.001
CD3+CD4+ lymphocytes	−0.121	−0.010
CD8+ lymphocytes	0.471*	−0.012
CD3−CD56+CD16+ lymphocytes	0.324*	0.011
CD8+CD57+ lymphocytes	**1**	0.173*
*Cytokines*		
IL-1*β*	0.121	0.695*
IL-6	0.113	0.540*
TNF-*α*	0.173*	**1**
INF-*γ*	0.044	0.706*

*P < 0.05.

**Table 4:** Multiple linear regression of CD8+CD57+ lymphocytes and TNF-*α* with MSPSS adjusted for socio-demographic characteristics.

	CD8+CD57+ lymphocytes (*β*)	TNF-*α* (*β*)
MSPSS score	−0.17*	−0.21*
Age	0.05	0.01
Gender male	−0.03	0.17
BMI	−0.07	−0.05
Alcohol drinkers	0.13	0.09

*P < 0.05.
